# Additive Influence of Extracellular pH, Oxygen Tension, and Pressure on Invasiveness and Survival of Human Osteosarcoma Cells

**DOI:** 10.3389/fonc.2013.00199

**Published:** 2013-07-29

**Authors:** Takao Matsubara, Gene R. DiResta, Shigeki Kakunaga, Dasen Li, John H. Healey

**Affiliations:** ^1^Department of Orthopaedic Surgery, Mie Graduate School of Medicine, Mie, Japan; ^2^Department of Chemical and Biomolecular Engineering, Polytechnic Institute of New York University, New York, NY, USA; ^3^Department of Orthopaedic Surgery, Osaka National Hospital, Kinki-Block Comprehensive Cancer Center, Osaka, Japan; ^4^Musculoskeletal Tumor Center, Peking University People’s Hospital, Beijing, China; ^5^Orthopaedic Research Laboratory, Department of Surgery, Memorial Sloan-Kettering Cancer Center, New York, NY, USA

**Keywords:** osteosarcoma, tumor microenvironment, hypoxia, acidity, elevated interstitial fluid pressure, carbonic anhydrase IX

## Abstract

**Background/Purpose:** The effects of chemical and physical interactions in the microenvironment of solid tumors have not been fully elucidated. We hypothesized that acidosis, hypoxia, and elevated interstitial fluid pressure (eIFP) have additive effects on tumor cell biology and lead to more aggressive behavior during tumor progression. We investigated this phenomenon using three human osteosarcoma (OS) cell lines and a novel *in vitro* cell culture apparatus.

**Materials and Methods:** U2OS, SaOS, and MG63 cell lines were cultured in media adjusted to various pH levels, oxygen tension (hypoxia 2% O_2_, normoxia 20% O_2_), and hydrostatic gage pressure (0 or 50 mmHg). Growth rate, apoptosis, cell cycle parameters, and expression of mRNA for proteins associated with invasiveness and tumor microenvironment (*CA IX, VEGF-A, HIF-1A, MMP-9*, and *TIMP-2*) were analyzed. Levels of CA IX, HIF-1α, and MMP-9 were measured using immunofluorescence. The effect of pH on invasiveness was evaluated in a Matrigel chamber assay.

**Results:** Within the acidic–hypoxic–pressurized conditions that simulate the microenvironment at a tumor’s center, invasive genes were upregulated, but the cell cycle was downregulated. The combined influence of acidosis, hypoxia, and IFP promoted invasiveness and angiogenesis to a greater extent than did pH, pO_2_, or eIFP individually. Significant cell death after brief exposure to acidic conditions occurred in each cell line during acclimation to acidic media, while prolonged exposure to acidic media resulted in reduced cell death. Furthermore, 48-h exposure to acidic conditions promoted tumor invasiveness in the Matrigel assay.

**Conclusion:** Our findings demonstrate that tumor microenvironmental parameters – particularly pH, pO_2_, and eIFP – additively influence tumor proliferation, invasion, metabolism, and viability to enhance cell survival and must be controlled in OS research.

## Introduction

Microenvironmental physical-chemical factors – such as pH, oxygen tension (pO_2_), and interstitial fluid pressure (IFP) – influence tumor proliferation, invasion, metabolism, and viability. Extracellular pH (pHe) and pO_2_ measured in malignant musculoskeletal tumors are lower than in benign tumors and normal tissues (1). In human osteosarcoma (OS), the eighth most common form of childhood cancer, a study of intraoperative IFP demonstrated a mean IFP of 35.2 ± 18.6 mmHg, which is significantly higher than IFP measured in adjacent normal tissues (2) and is similar to previously reported *in vivo* IFPs for renal cell and breast carcinomas (3). It is widely recognized that abnormal organization and function of tumor microvasculature create regions within the tumor that are acidotic, hypoxic, and have elevated interstitial fluid pressure (eIFP) (4, 5).

Osteosarcoma cell lines grown *in vitro* under elevated hydrostatic pressure, equivalent to mean *in vivo* IFP levels measured within central tumor regions, exhibit a more proliferative phenotype than cells grown under typical non-pressurized conditions (2). Tumor cells subjected to hypoxia tend to be more aggressive, displaying increased metastasis, invasion, and mutation (5); in OS specifically, higher levels of hypoxia inducible factor 1-alpha (HIF-1α) are associated with high-grade lesions and enhanced tumor cell growth *in vitro* (6). Acidosis, another common microenvironmental characteristic of malignant tumors, has been associated with increased rates of mutation (5) and influences expression of hypoxia-related genes (7, 8). While the acidic microenvironment is often attributed to the lactic acid produced through anaerobic metabolism induced by hypoxia, certain investigators have also implicated carbonic acid and, consequently, carbonic anhydrase IX (CA IX), as factors contributing to tumor acidosis (9, 10). The effects of each microenvironmental factor is also mediated through altered expression of additional biomarkers, such as hypoxia inducible factor 1 (HIF-1), vascular endothelial growth factor (VEGF), platelet derived growth factor (PDGF), matrix metalloproteinase (MMP) 2 and 9, and tissue inhibitor of metalloproteinase (TIMP) (9, 11–15). In addition, these factors have been shown to influence the effectiveness of conventional chemotherapy or radiation-based therapies (11).

While acidosis, hypoxia, and IFP frequently have been examined individually, we hypothesize that these microenvironmental factors exert additive effects on tumor cell biology and may lead to more aggressive or unexpectedly variable behavior during tumor progression and metastasis. Using a novel *in vitro* cell culture system in which cells can be grown under elevated hydrostatic pressure, we investigated the complex interactions of local pH, pO_2_, and hydrostatic pressure, individually and in combination, to identify the factor(s) that had the most profound effect on the growth of human OS cell lines and their expression of the proteins that contribute to metastatic potential. Further, because OS is a highly invasive cancer, we analyzed its invasiveness *in vitro* under acidic and hypoxic conditions. Ostensibly, control of the tumor microenvironment and the consequent altered expression of relevant proteins may allow suppression of aggressive tumor growth and the metastatic potential characteristic of high-grade malignant tumors.

## Materials and Methods

### Cell culture

We used three human OS cell lines (U2OS, SaOS2, and MG63) and, given the propensity for OS to metastasize to lung, we also included one lung carcinoma cell line (H1299) (American Type Culture Collection, Manassas, VA, USA). U2OS and SaOS2 lines were maintained in McCoy’s 5A media supplemented with 15% fetal calf serum (FCS); MG63 was grown in MEM media with 10% FCS; and H1299 was maintained in RPMI1640 media with 10% FCS.

### Microenvironmental factor combinatorial study

These experiments were performed using the OptiCell^®^ culture cassette system (Thermo Scientific, Austin, TX, USA), to which 0 or 50 mmHg hydrostatic pressure (gage) was applied in a manner that we have described previously (2). Briefly, the OptiCell^®^ chamber, which contains two parallel, gas-permeable, cell culture-treated polystyrene membranes that can support monolayer cell growth, was connected to a pressure bag system that was used to apply fluid pressure to the cell culture media. Use of this cell culture system within an incubator allows individual control of hydrostatic pressure, hypoxia, and media pH, to approximate the complex conditions that exist *in vivo*. The 0 mmHg gage pressure, comparable with conventional cell culture systems, served as the control pressure, while the 50 mmHg (gage pressure) was selected as the elevated hydrostatic pressure, corresponding to a level commonly seen in human and canine OS *in vivo* and consistent with our previous studies (2, 16, 17). The culture systems were placed into separate incubators, such that cassettes were exposed to hypoxic (2% O_2_, 5% CO_2_, and 93% N_2_) or normoxic (20% O_2_, 5% CO_2_, 75% N_2_) conditions. Both incubators were saturated to 100% humidity and temperature was maintained at 37°C. Cell culture media was prepared using two buffer systems, a 20 mM piperazine-*N,N*′-bis (2-ethanesulfonic acid) buffer to formulate the pH 6.5 and 6.8 media, and standard bicarbonate buffer to formulate the pH 7.4 and 7.1 media. pH was adjusted using 0.1 M HCl.

All cells were seeded at 1.5 × 10^5^ cells/cassette; separate cassettes were provided for each set of conditions. The experimental duration for the cell cycle and cell proliferation studies was 96 h total, comprising a 24-h, post-inoculation cell attachment phase, followed by 72 h of pressurization. The control conditions were pH 7.4, normoxic pO_2_, and 0 mmHg. To evaluate the long-term effects of acidic conditions on all cell lines, the cultures were acclimatized, or preconditioned, for 2 weeks in 75 cm^2^ flasks at pH 6.5, 6.8, 7.1, or 7.4. They were kept under normoxic pO_2_ levels and 0 mmHg hydrostatic pressure before transferral into the OptiCell^®^ cassettes and initiation of hydrostatic pressurization and/or hypoxia. At selected intervals, cells within the cassette were released from their membrane using trypsin, spun down, and counted to determine total viable cell count (TVCC). TVCC measured 24 h after seeding corresponded to the “0 h” time point in all studies. Net growth was expressed using the following ratio:

$$\text{Net growth}=\frac{\text{TVCC }(“\text{time point}”\text{ hour})}{\text{TVCC }(0\text{ hour})}$$

### Flow cytometry for cell cycle and apoptosis analyses

The H1299 cells, after 72 h of growth at 37°C, media pH 6.8, and 2% pO_2_, and under 500 mmHg of hydrostatic pressure, were collected, washed with cold PBS, and fixed in 70% methanol at 4°C. The U2OS cells, cultured for 72 h at 37°C; media pH 7.4, 7.1, 6.8, or 6.5; and 2% pO_2_, and under 50 mmHg of hydrostatic pressure, were similarly collected, washed, and fixed. Cells were pelleted and treated with 100 units/mL RNase, and DNA was fluorescently labeled with 0.05 mg/mL propidium iodide. The proportion of cells in the sub-G0/G1 phase and other cell cycle phases was evaluated with flow cytometry (FACSCaliber; Becton Dickinson, Bedford, MA, USA) and subsequent analysis with FlowJo software (Tree Star Inc., San Carlos, CA, USA).

### Quantitative real-time polymerase chain reaction gene expression assay

The gene expression of cells grown under different levels of extracellular pH, hypoxia, and hydrostatic pressure was determined after 24 h of exposure to the conditions. This time point was selected based upon previous investigations that identified 24 h as the time period after which maximal expression of adaptive response genes was observed (7, 8). The cDNA obtained from cells grown for 24 h under normal pH (7.4) or acidic pH (6.8); normoxia (20% O_2_) or hypoxia (2% O_2_); and normal or elevated hydrostatic pressure (0 or 50 mmHg), was assessed via QT-PCR for the relative expression of *CA IX, VEGF-A, HIF-1A, MMP-9*, and *TIMP-2* using the TaqMan Gene Expression Assays kit (Applied Biosystems, Foster City, CA, USA) and associated probes (*CA IX*: assay ID Hs00154208_m1; *VEGF-A*: assay ID Hs99999070_m1; *HIF-1A*: assay ID Hs00936368_m1; *MMP-9*: assay ID Hs00957555_m1; and *TIMP-2*: assay ID Hs00234278_m1). The manufacturer’s protocols were followed for the standardization, validation, and analysis assays, as previously described (18).

Total RNA for reverse transcription was extracted with Trizol (Invitrogen, Carlsbad, CA, USA) and treated with RNase-Free DNase I (QIAGEN, Valencia, CA, USA). cDNA was synthesized using a SuperScript II Wrst-Strand real-time polymerase chain reaction (RT-PCR) kit (Invitrogen). Gene expression was measured by quantitative RT-PCR (qPCR, MX4000, Stratagene, La Jolla, CA, USA) using 50 ng of rat cDNA and 2× TaqMan Universal PCR Master Mix (Applied Biosystems) with a one-step program (95°C for 10 min, 95°C for 30 s, and 60°C for 1 min for 50 cycles). The hypoxanthine phosphoribosyl-transferase (HPRT) gene was used as an endogenous normalizer. Duplicate samples without cDNA (no template control) for each gene showed no contaminating DNA.

### Immunofluorescence study of CA IX and HIF-1α

Cells were grown in the Nunc Lab-Tek II 4-well Chamber Slide System (Nalge Nunc International, Rochester, NY, USA); each well was seeded with 5 × 10^4^ cells. After culturing the cells at pH 7.4 for 24 h, the medium was replaced with pH 7.4, 6.8, or 6.5 media and cells were cultured in either 2% O_2_/98% N_2_ or 20% O_2_/80% N_2_ for an additional 12, 24, 36, and 48 h. The cells on the glass slides were washed with PBS and fixed with 4% paraformaldehyde.

An automated immunofluorescence staining protocol was performed with a Discovery XT processor (Ventana Medical Systems, Tucson, AZ, USA). Rabbit polyclonal antibodies were used for the primary incubation. The CA IX antibody (Santa Cruz, CA, USA, Cat. #sc-25599) was used at a concentration of 2.5 μg/mL, and the HIF-1α antibody (Chemicon, Temecula, CA, USA, Cat. #AB3883) was used at 12 μg/mL. Before the primary antibody incubation, the glass slides were blocked for 30 min in 10% normal goat serum, 2% BSA in PBS. Incubation with the primary antibody was 3 h, followed by 60 min incubation with biotinylated goat anti-rabbit IgG (Vector Labs, Burlingame, CA, USA, Cat. #PK6101) diluted 1:200. Normal rabbit IgG (5 μg/mL) was used as an appropriate isotype-negative control. The detection was performed with Streptavidin-HRP D (Ventana Medical Systems), followed by incubation with either Tyramide-Alexa Fluor 488 (Invitrogen, Cat. #T20922) for CA IX or with Tyramide-Alexa Fluor 568 (Invitrogen, Cat. #T20914) for HIF-1α.

Cells were visualized using fluorescence microscopy (ZEISS Axioplan2 Imaging). ZEISS AxioVision (ver. 4.6.3.SP1) and Adobe Photoshop (Adobe Systems Inc., San Jose, CA, USA) were used to analyze the quantity of each protein level. Fluorescence photographs were taken with light exposure times of 400 ms for CA IX and 500 ms for HIF-1α; two photographs were taken in each group. Fluorescence was quantified with Photoshop, using a previously published method of evaluating luminosity (19). Histograms were derived from the pixel density average from both photographs.

### Invasion assay

An invasion assay was performed on the OS cell lines using the Biocoat Matrigel Invasion Chamber (Becton Dickinson). After culturing the OS cells in pH 7.4 or 6.8 media for 48 h (20% pO_2_, 0 mmHg), 5 × 10^4^ cells of U2OS, 15 × 10^4^ cells of MG63, or 5 × 10^4^ cells of SaOS2 were added to the invasion chamber and cultured for 24 h in pH 7.4 media under 2 or 20% pO_2_. FCS was used as the chemoattractant; cells incubated without a chemoattractant served as controls. After 24 h, non-invasive cells were removed from the upper surface of the membrane with a cotton swab. The invasive cells on the lower surface of the membrane were fixed with 4% methanol, stained with toluidine blue, and counted in four separate areas with an inverted microscope (ZEISS, Dublin, CA, USA). An “invasion index” was computed using the manufacturer’s analysis protocol, in which the invasion index was defined as the quotient of the percent cell invasion in a test chamber divided by the percent cell invasion in a control chamber.

### Statistical analysis

Student’s *t* test was used to assess the significance of any differences between the results of each condition relative to control. Each data point shown in the figures is the mean ± standard error (*n* = 3 cassettes/condition), and significance is defined as *P* < 0.05.

## Results

### Cell proliferation and flow cytometry analysis on U2OS

**Figure 1:**
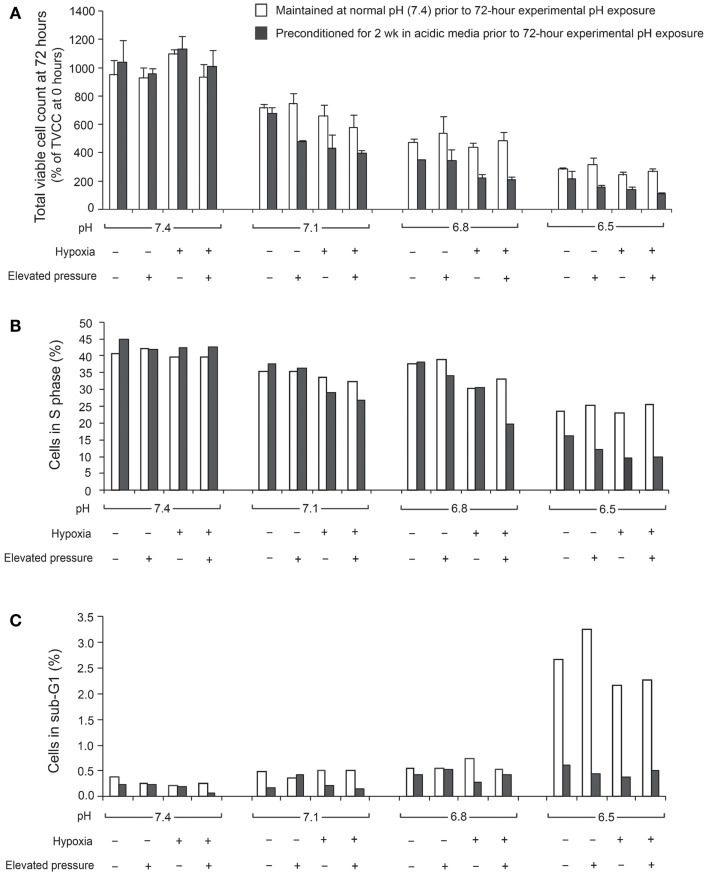
**Total viable cell count (TVCC) ratios of U2OS cells after 72 h exposure to different pH levels: (A) under various extracellular pH, pO_2_, and hydrostatic fluid pressures; (B) in S phase; (C) in Sub-G0/G1 phase**. TVCCs measured 24 h after seeding corresponded to the “0 h” time point in all studies. Net growth at 72 h was expressed relative to the initial count at 0 h (TVCC ratio). Cells were grown with and without a 2-week preconditioning phase in acidic medium prior to the experimental pH exposure period.

The ratio of TVCC at 72 h to the TVCC at 0 h (24 h after seeding) was used to calculate net growth of U2OS cells at various pH, pO_2_, and hydrostatic pressure levels after 2 weeks of preconditioning at acidic pH levels or normal pH (Figure 1A). The percentage of cells in S phase and in the sub-G0/G1 phase, which correlate with DNA replication activity and apoptosis rate, respectively, are presented in Figures 1B,C. The findings suggest that U2OS tumor growth at 72 h decreased as pH decreased (Figure 1A). Previous studies using pH 7.4 media showed that U2OS growth was enhanced under hypoxia and elevated hydrostatic pressure (11); however, our data suggest that hypoxic and hydrostatic pressure levels inhibited growth of U2OS cells grown in acidic media (Figure 1A). In addition, as pH decreases, the growth of cells exposed to either hypoxic or elevated hydrostatic pressure conditions for a 2-week period was further decreased, while the apoptosis rate after 2 weeks’ culture at pH 6.5 was not increased relative to cells cultured at pH 6.5 for 72 h. Under acidic conditions, the cells’ S phase decreased (Figure 1B); the slowing of the cell cycle in U2OS by acidic media was evident during both the 72-h and the 2-week growth periods. Apoptosis, however, was not promoted by 2 weeks of exposure to acidic media (Figure 1C; dark bar).

**Figure 2:**
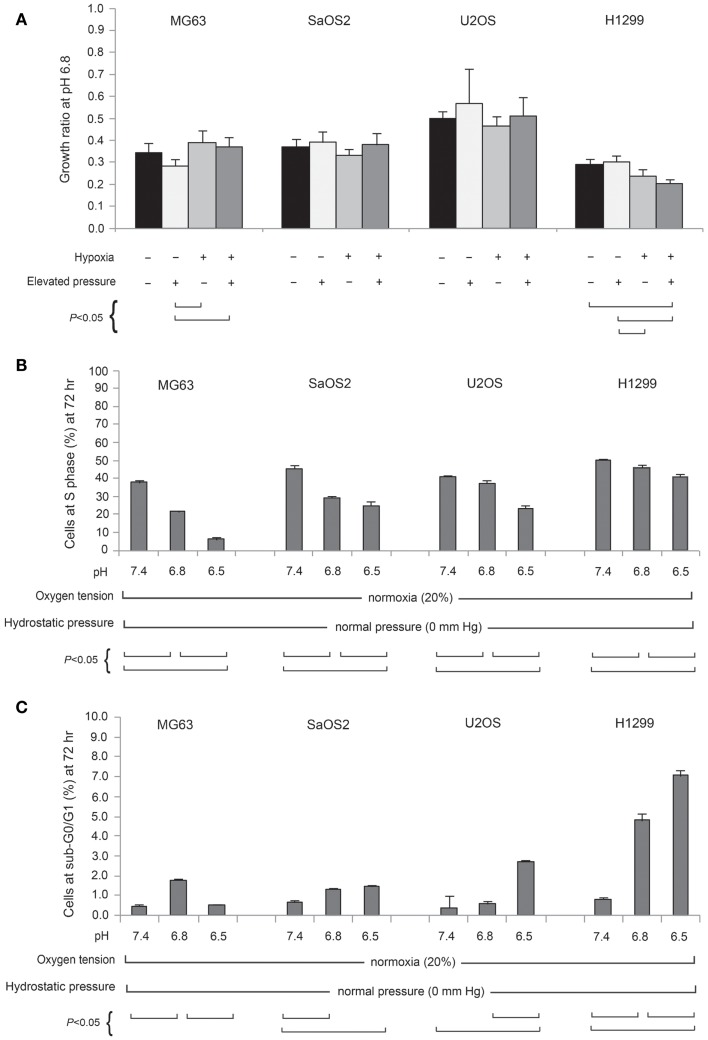
**(A)** Normalized total viable cell counts (TVCC) of cell lines cultured for 72 h at pH 6.8, 20% (normoxic) or 2% (hypoxic) pO_2_, and 50 mmHg hydrostatic pressure conditions, and at pH 6.5, normoxia, and 0 mmHg hydrostatic pressure. Each cell line bar is normalized to its respective TVCC after 72 h culture at pH 7.4, normoxia, and 0 mmHg hydrostatic pressure. Percentage of cell lines in S phase **(B)** and sub-G0/G1 phase **(C)** at pH 7.4, 6.8, and 6.5.

Figure 2A presents the TVCC ratio of all tumor cells after 72 h under pH 6.8, with: 20% pO_2_, and 0 mmHg hydrostatic pressure; 20% pO_2_, and 50 mmHg hydrostatic pressure; 2% pO_2_ and 0 mmHg hydrostatic pressure; and 2% pO_2_ and 50 mmHg hydrostatic pressure. Comparison with cells grown under pH 7.4 and 20% pO_2_ (control condition), suggests that the TVCC ratio of all tumor cell lines is reduced in acidic media. At pH 6.8, the growth of all tumor cells is not promoted by hypoxia or elevated hydrostatic pressure. For all tumor cell lines, no significant difference was observed in cell cycle and apoptosis rates of cells preconditioned in acidic media and subsequently grown in acidic media, whether under elevated hydrostatic pressure, hypoxia, or a combination of hypoxia and hydrostatic pressure (data not shown). On the other hand, acidosis was associated with changes in cell cycle (Figure 2B) and apoptosis rates (Figure 2C) compared with pH 7.4 neutral condition. As seen in Figure 2C, with the exception of the H1299 cell line, apoptosis rates in cells grown under acidic conditions were not strongly affected as extracellular pH decreased (sub-G0/G1 phase was moderately affected in U2OS cells at pH 6.5). Further, cell counts performed after trypan-blue staining in both supernatants and detached monolayer cells before and after a 72-h incubation in either pH 7.4 or 6.8 culture medium did not reveal significant cell death. As for apoptosis, OS cell lines were relatively resistant to the influence of acidic media compared with the H1299 lung cancer cell line (Figure 2C).

### Quantitative RT-PCR assay for *CA IX*

**Figure 3:**
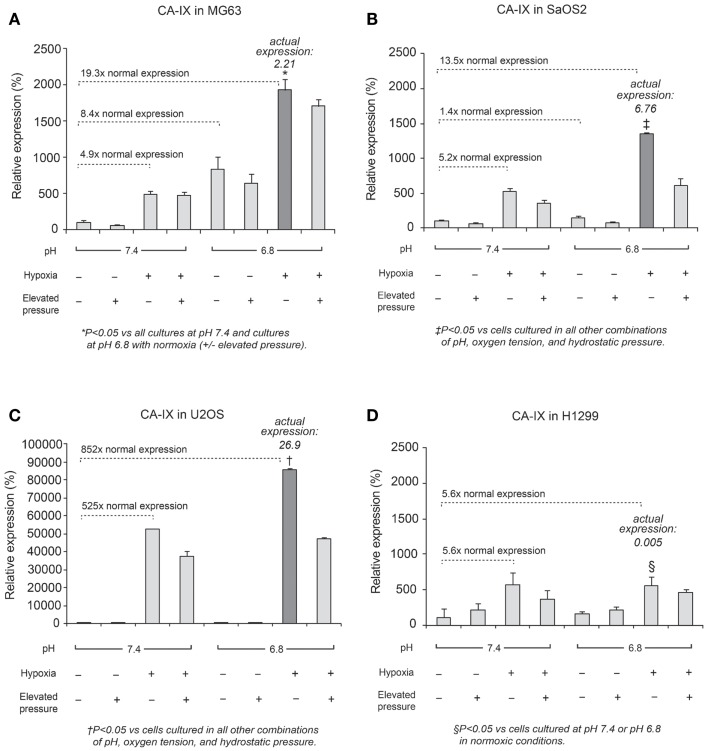
**Relative expression levels of CA IX mRNA, determined via quantitative real-time PCR, in four tumor cell lines: (A) MG63, (B) SaOS2, (C) U2OS, and (D) H1299**. The dark columns highlight the acidic-hypoxic-non-pressurized conditions, which elicited the highest expression of *CA IX* in the osteosarcoma cell lines. The *italic* number in each panel represents the real *CA IX* expression levels (normalized to *HPRT*) in these growth conditions. The normalized *CA IX* expression level in U2OS (26.9) was the highest among osteosarcoma cell lines. *CA IX* expression in H1299 (0.005) was very weak compared with osteosarcoma cells. The gene expression levels for select microenvironmental combinations, presented as a multiple of the expression levels under neutral/control conditions, are indicated by the dotted lines. In MG63, hypoxia increased *CA IX* expression 4.9-fold and acidic pH increased expression 8.4-fold, while the combined effect of hypoxia and acidic conditions elevated *CA IX* expression by a factor of 19.3. Hypoxia and acidic conditions elevated *CA IX* expression 852-fold in U2OS and 13.5-fold in SaOS2, while combined conditions did not promote increased *CA IX* expression in H1299.

In all cell lines, *CA IX* mRNA expression was higher under hypoxic conditions than under normoxic conditions (Figure 3), and was related to HIF-1α expression, as previously reported (9, 20). Furthermore, in OS cell lines, *CA IX* mRNA was highly expressed under both acidosis and hypoxia. Hydrostatic pressure reduced *CA IX* gene expression in OS cells; however, in acidic and hypoxic conditions without elevated hydrostatic pressure, *CA IX* was strongly expressed compared to normoxic and normal pH conditions (Figure 3; dark bar). The actual expression of *CA IX* in OS cells was higher than in H1299 lung cancer cells (Figure 3). Compared with typical cell culture conditions (pH 7.4, 20% oxygen, and 0 mmHg pressure, which we considered to be the “neutral” condition), the combinations of altered microenvironmental conditions increased gene expression by the factors shown in the figure. Specifically, in MG63 cells, hypoxia elevated *CA IX* expression 4.9-fold over the neutral condition, and acidic pH elevated expression 8.4-fold, while the combined effect of hypoxia and acidic condition elevated *CA IX* expression by a factor of 19.3. The combination of hypoxia and acidic pH elevated *CA IX* expression 852-fold in U2OS cells and 13.5-fold in SaOS2, while these conditions failed to promote significantly elevated *CA IX* expression in H1299.

### Quantitative RT-PCR assay for *VEGF-A*

**Figure 4:**
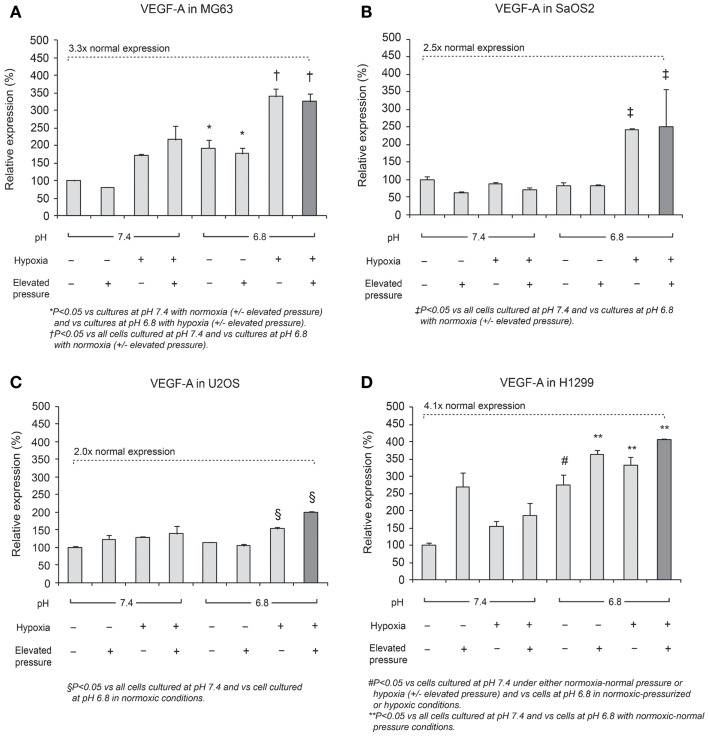
**Relative expression levels of VEGF-A mRNA, determined via quantitative real-time PCR, in four tumor cell lines: (A) MG63, (B) SaOS2, (C) U2OS, and (D) H1299**. The dark columns show the combined conditions of acidic pH, hypoxia (2% O_2_), and elevated hydrostatic pressure (in which *VEGF-A* was the most highly expressed). In all cell lines, *VEGF-A* was highly expressed in the combination of low pH, hypoxia, and high pressure. The dotted lines highlight gene expression levels under altered microenvironmental conditions, presented as a multiple of the levels under neutral conditions. *VEGF-A* expression was 3.3× higher in MG63 cells, 2.5× higher in SaOS2, 2.0× higher in U2OS, and 4.1× higher in H1299 cells.

Expression of *VEGF-A* mRNA is shown in Figure 4. The combined effect of acidosis, hypoxia, and elevated hydrostatic pressure (dark bar in the display for each cell line) increased *VEGF-A* mRNA expression significantly compared with control conditions (*P* < 0.05). The combination of acidic pH, hypoxia, and high hydrostatic pressure elicited *VEGF-A* levels that were 3.3-fold higher than the neutral conditions in MG63 cells and 4.1-fold higher than the control conditions in H1299 cells. These findings suggest that *VEGF-A* expression is additively influenced by combinations of altered microenvironmental factors.

### Quantitative RT-PCR assay for *HIF-1A*

**Figure 5:**
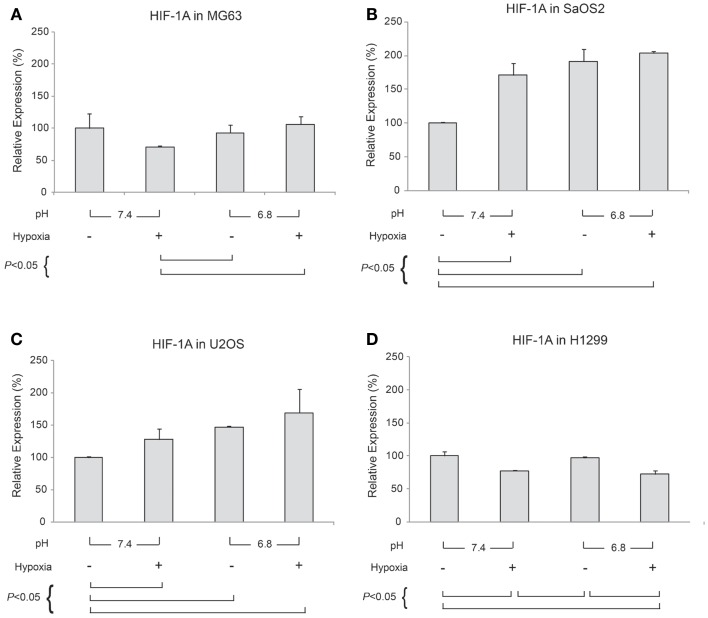
**Relative expression levels of *HIF-1A* mRNA, determined via quantitative real-time PCR, in four tumor cell lines: (A) MG63, (B) SaOS2, (C) U2OS, and (D) H1299**. All graphs show mRNA expression under normal hydrostatic pressure, and indicate statistically significant differences between different combinations of pH (7.4 or 6.8) and either normoxia (20% O_2_) or hypoxia (2% O_2_).

Generally, HIF-1α is rapidly degraded by proteasomal degradation. During low oxygen tension conditions (2% pO_2_), this degradation is inhibited, leading to increased *HIF-1A* (21). In contrast, our findings demonstrated that *HIF-1A* was increased by hypoxic conditions in SaOS2 and U2OS cell lines as previously reported (Figure 5). With respect to *HIF-1A* mRNA levels, elevated IFP did not affect the expression of *HIF-1A* (elevated IFP conditions are not included in Figure 5). Furthermore, low pH led to increased *HIF-1A* without stimulation by hypoxia in SaOS2 and U2OS. The combinatorial effect of hypoxia and acidity did not promote further *HIF-1A* expression in MG63 and H1299, while these microenvironmental parameters additively promoted mRNA of the hypoxia-induced factors *CA IX* and *VEGF-A*.

### *MMP-9* and *TIMP-2* expression

**Figure 6:**
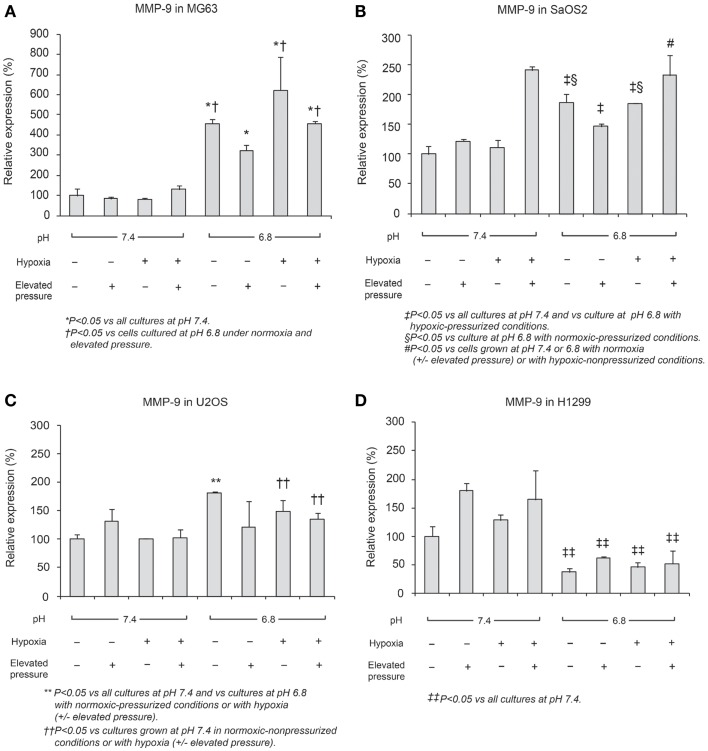
**Relative expression levels of MMP-9 mRNA, determined via quantitative real-time PCR, in four tumor cell lines: (A) MG63, (B) SaOS2, (C) U2OS, and (D) H1299**. The number on each graph is the maximum level of the target mRNA/HPRT in each cell line.

**Figure 7:**
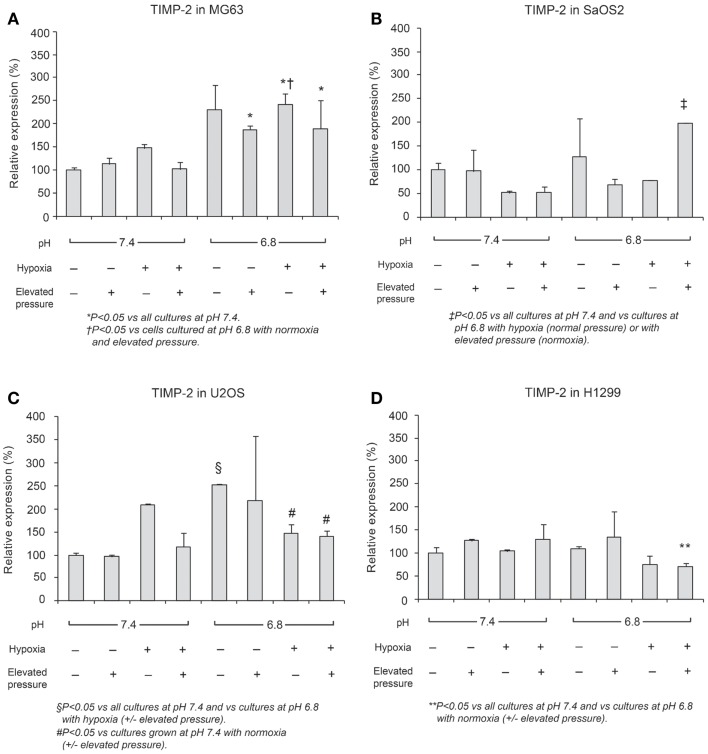
**Relative expression levels of TIMP-2 mRNA, determined via quantitative real-time PCR, in four tumor cell lines: (A) MG63, (B) SaOS2, (C) U2OS, and (D) H1299**.

In Figures 6 and 7, mRNA expression levels of *MMP-9* and *TIMP-2* are presented. The level of *MMP-9/HPRT* (the endogenous control for mRNA expression) was 5.57 in MG63 cells, 0.36 in SaOS2, 71.9 in U2OS, and 2.74 in H1299 cells. *MMP-9* was more highly expressed in MG63 and U2OS cells than in SaOS2 and H1299 cells. In MG63 and SaOS2, *MMP-9* expression was higher in acidic conditions than in control conditions (Figure 6). In MG63 cells, *TIMP-2* was more highly expressed in acidic media than in normal pH media (Figure 7; *P* < 0.05), but in the other cell lines, acidic growth conditions didn’t increase the expression of *TIMP-2*. Elevated hydrostatic pressure promoted greater expression of *MMP-9* in SaOS2 and H1299 cells. For *MMP-9* and *TIMP-2*, acidic media was the most influential factor for two (MG63 and SaOS2) of three OS cell lines. Elevated hydrostatic pressure and acidic media each enhanced mRNA expression of *MMP-9* in tumor cells.

### Immunofluorescence studies: CA IX and HIF-1α

**Figure 8:**
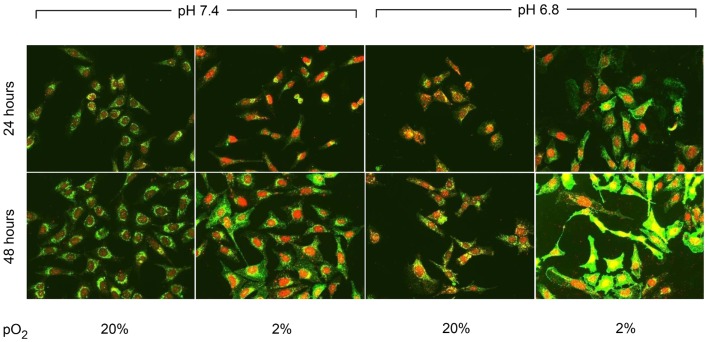
**Immunofluorescent staining demonstrated the changes in CA IX (green) and HIF-1α (red) expression at 24 and 48 h of incubation in varying microenvironmental conditions**. CA IX expression was highly promoted by the combination of acidic pH and hypoxia.

**Figure 9:**
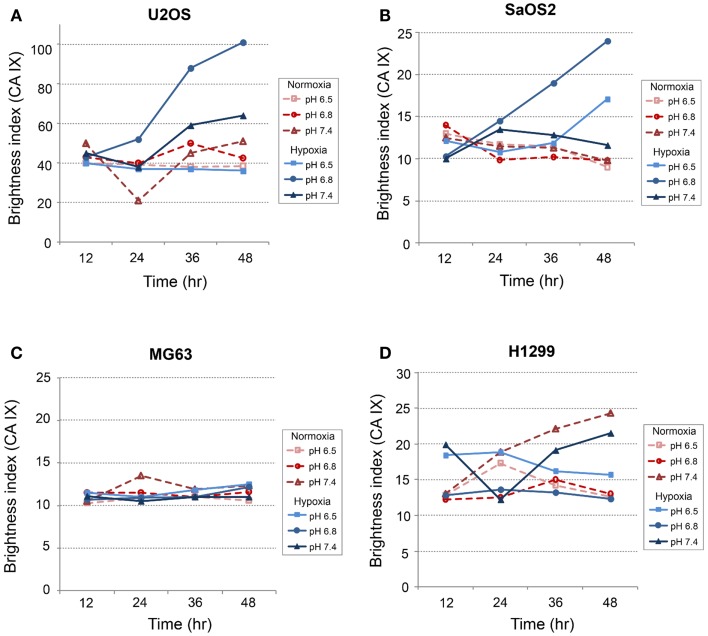
**Expression level of CA IX in (A) SaOS2, (B) U2OS, (C) MG63, and (D) H1299 cells**.

**Figure 10:**
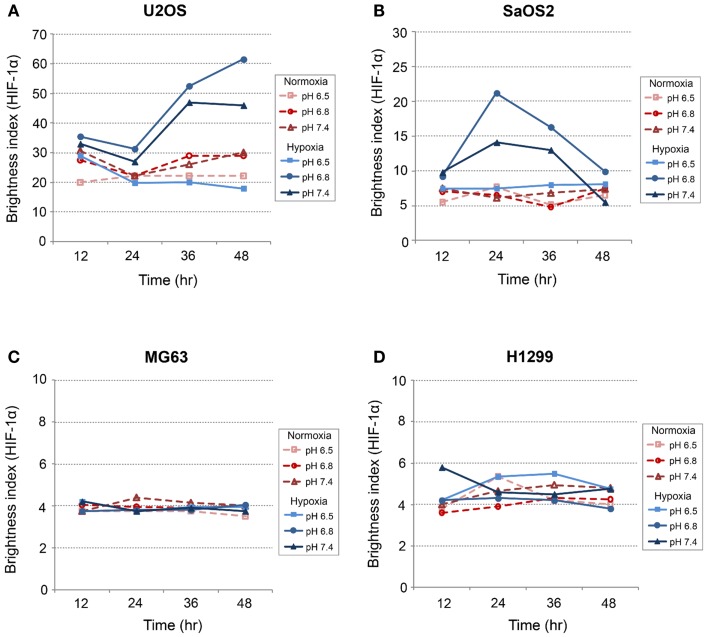
**Expression level of HIF-1α in (A) SaOS2, (B) U2OS, (C) MG63, and (D) H1299 cells**.

*CA IX* and *HIF-1A* mRNA expression was affected more by acidic pH and hypoxia than by elevated IFP. The effects of pH and hypoxia on CA IX and HIF-1α protein level expression and their relationship to tumor invasiveness were also studied using immunofluorescence with *in vitro* cell culture. The immunofluorescence images of colocalized CA IX and HIF-1α in U2OS are presented in Figure 8. The immunofluorescence brightness indices corresponding to CA IX and HIF-1α expression in each cell line are graphically represented in Figures 9 and 10. U2OS cells expressed CA IX consistently under normal pH and normoxic conditions, but exposure to acidic media and hypoxic conditions resulted in additive upregulation of CA IX in U2OS and SaOS2 cells (Figures 7 and 8). These data were corroborated by the upregulation of *CA IX* mRNA expression in the quantitative RT-PCR analysis (Figure 3). On examining the correlation between *HIF-1A* mRNA expression and HIF-1α, the combinatorial effect of low pH and hypoxia promoted HIF-1α expression in U2OS and SaOS2, while the exposure to these microenvironmental conditions did not upregulate expression of *HIF-1A* mRNA. The expression of *HIF-1A* mRNA and HIF-1α were not parallel under the combined acidotic and hypoxic condition. The minimal differences in CA IX immunofluorescence staining between MG63 and H1299 cells in various growth conditions (Figures 9C,D) were also consistent with the level of mRNA expression data from quantitative RT-PCR (Figure 3). The expression of CA IX and HIF-1α were generally parallel in all cell lines (Figures 9 and 10).

### Invasion assay

The invasion indices for MG63, SaOS2, and U2OS cells are shown in Table 1. The invasion index of U2OS cells was higher in acidic conditions, with or without hypoxia, than in the normal pH–hypoxic and normal pH–normoxic conditions (*P* < 0.05). The invasion index of SaOS2 cells was not upregulated by either acidic or hypoxic conditions.

**Table 1:** **The invasion index of three osteosarcoma cell lines**.

Cell line	Media conditions			
	pH 7.4,20% O_2_	pH 6.8,20% O_2_	pH 7.4,2% O_2_	pH 6.8,2% O_2_
MG63	7.38 ± 1.3	8.26 ± 1.3	7.69 ± 4.0	16.65 ± 1.7*
SaOS2	14.20 ± 5.0	9.28 ± 3.7	17.60 ± 5.8	9.04 ± 4.2
U2OS	9.69 ± 5.8	31.72 ± 4.2^†^	16.53 ± 5.9	32.03 ± 6.6^†^

*For MG63: P < 0.05 for acidic-hypoxic conditions vs. normal pH and acidic-normoxic conditions.

*^†^For U2OS: P < 0.05 for acidic-hypoxic and acidic-normoxic conditions vs. pH 7.4 (with and without hypoxia)*.

## Discussion

*In vitro* tumor cell culture conditions typically lack the elevated pressure, hypoxia, and acidosis frequently observed in solid tumors *in vivo* (11, 12). Heretofore, experimental reports have examined these physical-chemical factors in isolation, not in concert. To evaluate the combinatorial effects of microenvironmental factors on the growth of U2OS, SaOS2, MG63, and H1299 cell lines, we used our novel cell culture system to individually control these parameters and to independently simulate various combinations of elevated pressure, hypoxia, and acidity that occur *in vivo*. We demonstrated that these factors exert independent and additive effects on both cellular growth and the expression of genes that regulate malignant phenotype. These findings suggest a need to control for each of these physical-chemical environmental conditions in future cell culture and chemosensitivity studies in the development of future therapies for OS.

In U2OS cells, continuous exposure to acidic conditions reduced proliferation and promoted invasiveness but did not significantly influence apoptosis. Cells cultured in acidic media for 72 h under hypoxic or elevated hydrostatic pressure did not evince tumor cell proliferation as was observed in cells cultured under normal pH (2). However, continuous exposure to acidic conditions for 2 weeks under hypoxic or elevated hydrostatic pressure mediated cell cycle progression and downregulated cell growth. We also observed that U2OS cell cycling slowed with decreasing extracellular pH (pH 7.1–6.8), while pH below 6.8 induced apoptosis (Figures 1B,C). This observation might be consistent with *in vivo* observations of the center of human OS tumors, which tend to be necrotic and acidic (1). Our data demonstrate the complex interactions of microenvironmental factors on tumor physiology and indicate that pH has the most profound influence on cell progression.

In our cell proliferation studies, neither hypoxia nor elevated hydrostatic pressure promoted proliferation in acidic media. Our studies demonstrated that mildly acidic pH downregulated tumor growth, even under conditions of hypoxia or elevated pressure. In our OS cell lines, although more acidic conditions were not associated with cellular proliferation, they also were not associated with necrosis or apoptosis. Hence, we can hypothesize from the proliferation study that the survival of OS cells in acidic conditions may be achieved through reduced rates of cellular metabolism, adaptation to acidity, or migration to regions with normal pH, pO_2_, and IFP.

On the other hand, *VEGF-A* mRNA was expressed in acidic, hypoxic, and elevated pressure conditions. A previous study by our group demonstrated that *VEGF-A* in cells cultured at normal pH was not upregulated by elevated hydrostatic pressure (13). However, we speculate that the combination of hypoxia with low pH and elevated IFP, conditions that occur within a tumor’s central region or in regions away from blood vessels *in vivo*, may induce angiogenesis through increased expression of VEGF.

Studies have reported CA IX expression in several carcinomas, such as renal cell (22), colorectal (23), non-small cell lung (24, 25), cervical (26), bladder (27), nasopharyngeal carcinoma (28), breast (29), and soft tissue sarcoma (30), but it is absent from most normal tissues. CA IX has long been examined as a marker of tumor hypoxia, and is quickly and extensively upregulated under hypoxic conditions (31, 32). Furthermore, a few published reports have demonstrated that extracellular acidosis elevates CA IX expression in tumor cells (7, 8). CA IX mediates the transport of an intracellular H^+^ to the extracellular space to maintain homeostasis in tumor cells (11). Thus, we hypothesized that CA IX is not only a biomarker for hypoxia, but also serves as a marker of acidosis in tumors.

In our study, *CA IX* mRNA was highly expressed under hypoxia in all OS tumor cell lines, and the combination of acidosis and hypoxia further promoted *CA IX* mRNA expression. Hydrostatic pressure downregulated the effect of hypoxia in all OS cell and lung cancer cell lines, and acidosis induced upregulation of *CA IX* in all OS cell lines. But *CA IX* was more highly expressed in cells cultured in acidic–hypoxic–non-pressurized media conditions than in OS cells cultured under normal pH and normal oxygen media (Figure 3). This *CA IX* mRNA finding was confirmed by immunofluorescence study (Figure 8). CA IX expression was highest in the combination of acidic (pH 6.8) and hypoxic growth conditions for the U2OS and SaOS2 OS cell lines (Figures 8 and 9), as indicated by the mRNA study (Figure 3). CA IX in these OS cell lines was regulated by the combination of hypoxia and acidity. Our study demonstrated that CA IX, long regarded as a hypoxia-induced factor, was upregulated by the complex conditions of hypoxia and acidity, although these combined conditions did not promote increased expression of *HIF-1A* mRNA (Figure 5). It has been reported that extracellular acidosis elevates CA IX expression in tumor cells, even in normoxic conditions (7, 8, 49). This is further supported by studies of the von Hippel–Lindau tumor suppressor protein (pVHL), the main negative regulator of HIF-1, which can downregulate CA IX (obviously as a direct HIF-1 target) (33, 34). HIF is degraded in conditions of normal oxygen tension by pVHL, but is stabilized by hypoxia. CA IX, as part of the hypoxic acidification mechanism, might facilitate the nucleolar sequestration of pVHL and activation of HIF, which has been described as a pH-dependent mechanism that may serve a protective role in reoxygenated cells (35). In this case, HIF-mediates increases in the level and activity of CA IX, resulting in enhanced acidification, and might create a feedback loop that leads to prolonged HIF activation. As a result of this prolonged HIF activation, CA IX would be increased in hypoxic-acidic conditions without upregulation of *HIF-1A* mRNA. CA IX is reported to have a major role in regulating H^+^ flux, while blockade of CA IX results in increased cell death (9). The immunofluorescence study demonstrates that this is one mechanism for acidic adaptation and suggests that CA IX may be a potential target for therapy and a marker for OS (51).

Physical-chemical conditions affect markers of aggressive behavior. In acidic–hypoxic–hydrostatically pressurized media conditions, the TVCC at 72 h was reduced. However, our studies demonstrated that the mRNA of *MMP-9*, a marker of invasive potential, was upregulated in acidic–hypoxic–hydrostatically pressurized media conditions for U2OS and MG63 cells (Figure 6). Based on the *HPRT-*normalized levels of *MMP-9*, we demonstrated that this metalloprotease was more highly expressed in MG63 and U2OS cells than in SaOS2 and H1299 cells. Increased levels of *MMP-9* under acidic-hypoxic conditions may have contributed to the increased invasive activity observed in the MG63 and U2OS cell lines (Table 1), and this metastatic potential may be further influenced by acidosis- and hypoxia-associated changes in interstitial pressure (36, 37). Although invasive potential under hydrostatic pressure was not specifically examined in this study, our findings do suggest that elevated pressure promotes VEGF-associated angiogenesis.

The high metabolic and growth rates of tumor cells deplete local supplies of oxygen and nutrients, promoting a switch from aerobic to anaerobic metabolism. This contributes to extracellular acidosis and slows down the cell cycle, as our proliferation study revealed. With further growth, the tumor’s IFP increases; eventually local hypoxia, acidic pH, and high hydrostatic pressure conditions become severe in the tumor center or areas distant from the tumor vasculature. The combination of these factors exert multiple biological effects, inducing CA IX to adapt to significantly acidic conditions; upregulating angiogenesis via VEGF to improve hypoxia and/or low nutrition; and increasing expression of MMP-9 to enhance tumor cell invasion and migration to new microenvironments conducive to further growth.

Overall, U2OS most consistently responded to microenvironmental changes with increased invasive potential and altered expression of biomarkers. The higher invasive potential of U2OS relative to the other OS cell lines in our study is consistent with prior reports (15, 38); MMP-9, one marker of invasiveness, is consistently expressed in U2OS as well as human OS biopsy tissue (38). With respect to expression of both *MMP-9* and *TIMP-2*, MG63 exhibited the most pronounced upregulation response to an *in vitro* microenvironment that most resembled *in vivo* tumor growth conditions; both of these biomarkers are consistently expressed in OS biopsy samples (38). Across the individual OS cell lines in our studies, some level of variation in the phenotypic responses to microenvironment alteration was evident and may be attributable to patient-specific genotypic variation and differences in metastatic potential among the tumors from which the cell lines were derived (50). Reapplication of microenvironmental parameters that typify OS tumors (hypoxia, high IFP, acidosis), achieved through our novel cell culture apparatus, can facilitate the restoration of previous *in vivo* phenotypic characteristics, including differences in tumor cells innate genotypic ability to respond to evolving microenvironmental conditions *in vivo*.

Since cell-capillary diffusion distances are greater in tumors, diffusive coupling is poor and can compound extracellular acidosis (11, 48). It is widely understood that CA IX and VEGF expression levels are influenced by levels of HIF-1, which promotes tumor invasiveness. In our study, genes and proteins associated with angiogenesis and invasive potential were also influenced by the additive effects of increased acidosis, hypoxia, and IFP. The tumor cells exposed to severe acidosis exhibited a tendency toward apoptosis or cell cycle arrest, an effect that was compounded by hypoxia and/or elevated hydrostatic pressure. Tumor cells that migrate to an anatomic location with neutral pH, such as a lung, respond to the elevated hydrostatic pressure with enhanced growth. A tumor’s microenvironment may synergistically induce metabolic and phenotypic alterations that control the tumor’s survival by regulating cell proliferation, angiogenesis, and invasiveness.

A number of factors limit the extrapolation of our results to *in vivo* tumor growth and invasiveness. First, tumor cells *in vitro* receive complete nutrition from cell culture media, in contrast to the compromised delivery of nutrients by the altered vasculature within a tumor *in vivo*, particularly within the most central regions. Second, because the OptiCell chamber membrane is not compatible with immunofluorescence staining techniques, the effects of elevated pressure could not be evaluated via immunofluorescence studies. Additionally, our culture system allowed application of only a single hydrostatic pressure and could not replicate the *in vivo* decreasing pressure gradient that extends from a tumor’s central region to peripheral regions (39). This gradient may be responsible for the differential, location-dependent expression of angiogenic and hypoxic biomarkers that has recently been reported (40). Third, we did not employ any three-dimensional culture systems to examine the effects of microenvironmental factors on spheroidal tumor cell aggregates, which recapitulate *in vivo* tumor growth more closely; such three-dimensional cellular growth influences cell metabolism and, combined with hypoxia, can increase invasive potential in MG63 cells (41, 42). Finally, as with any *in vitro* system for the study of malignant tumors, the lack of an extracellular matrix (ECM) precludes the examination of any factors which could contribute further additive effects to tumor cell growth and metastatic potential; in the case of OS, the influences of MMP/TIMP interactions and ECM-associated growth factors on invasive potential have been examined but have not yet been fully elucidated (14, 15, 43–47). However, despite these limitations, this study suggests that tumor microenvironmental factors must be considered in aggregate and that the control of tumor microenvironmental factors, and/or expression of CA IX and VEGF, should be among the important targets for examination in the development of future therapies for malignant tumors.

## Conflict of Interest Statement

The authors declare that the research was conducted in the absence of any commercial or financial relationships that could be construed as a potential conflict of interest.
